# Characterization of Epigenetic and Molecular Factors in Endometrium of Females with Infertility

**DOI:** 10.3390/biomedicines10061324

**Published:** 2022-06-04

**Authors:** Giedrė Skliutė, Raminta Baušytė, Diana Ramašauskaitė, Rūta Navakauskienė

**Affiliations:** 1 Department of Molecular Cell Biology, Institute of Biochemistry, Life Sciences Center, Vilnius University, Saulėtekio Av. 7, LT-10257 Vilnius, Lithuania; giedre.skliute@bchi.stud.vu.lt (G.S.); raminta.bausyte@santa.lt (R.B.); 2 Centre of Obstetrics and Gynaecology, Institute of Clinical Medicine, Faculty of Medicine, Vilnius University, Santariškiu˛ St., LT-08661 Vilnius, Lithuania; diana.ramasauskaite@santa.lt

**Keywords:** reproduction, reproductive disorders, reproductive pathology, infertility, endometrium, endometrial tissue

## Abstract

Infertility is one of the most rapidly increasing global health concerns of the 21st century. Embryo quality and endometrial thickness and receptivity are the main factors for successful embryo implantation and pregnancy development. Nevertheless, until now, there has been a lack of understanding about the regulation of human endometrium function and its structure. This raises the demand for more research of the human endometrium in these fields. In our study, we analyzed the genetic and epigenetic changes of endometrial tissue’s samples isolated from females admitted for treatment due to male infertility and females diagnosed with reproductive pathologies, who are preparing for assisted reproductive technologies procedures. Using real-time polymerase chain reaction method, we demonstrated that endometrium of females with reproductive pathology has significantly upregulated decidualization related genes *HAND2*, *MUC1*, *CSF2*, increased expression of angiogenesis related gene *PDGFA*, and increases of overall immune response and inflammation-related genes expression with significant changes of *RELA* and *CXCL10* genes expression. Females with reproductive pathology have altered endometrium epigenetic regulation since expression of miRNAs—specifically, miRNA-34a, miRNA-223, and miRNA-125b—is lower in endometrium of females with reproductive pathology. Our findings suggest that the potential changes in genetic and epigenetic profile of endometrium from females with reproductive pathology could enrich the knowledge in the field of core biological knowledge and treatment of reproductive impairments.

## 1. Introduction

Failure of embryo implantation is one of the major limiting factors in successful pregnancy establishment and development after spontaneous conception and assisted reproductive technologies procedures. Implantation involves the synchronized dialogue between an implantation-competent blastocyst (a blastocyst is a human embryo that is five or six days old) and a receptive endometrium. Endometrial receptivity is defined as a limited timeframe which combine complex process that provides the embryo with the opportunity to attach, invade and develop. In this way, one of determinants of successful implantation and pregnancy development is the endometrial receptivity [1,2,3,4]. The time period when the endometrium is receptive to the embryo implantation is called the window of implantation that usually occurs during 6 days after the peak in luteinizing hormone levels, between days 19 and 21 of the cycle [5,6]. After implantation, the embryo damages the luminal epithelium, and the stromal cells surrounding the implanted embryo are decidualized, and this process is regulated by the hormone progesterone [7,8]. Decidualization includes the emerging of new types of endometrial cells, epithelioid cells, which provide nutrition and support for the implanting blastocyst until the placenta is fully formed [9,10].

Fertility disorders affect 8–12% of reproductive age couples worldwide. Infertility diagnosis and treatment methods carry numerous drawbacks [11,12,13,14]. For example, it is reported, that infertility has a strong negative effect on the quality of life. It is counted that more than half of the infertile females indicate a different degree of disorders, such as-anxiety, social dysfunction, and depression [15]. Studies show that even after the unprecedented global COVID-19 pandemic, which caused economic and social uncertainty, the infertility factor remains significant and has even increased [16].

In seeking to optimize the chances of people with fertility problems achieving parenthood and obtaining a healthy offspring, more studies should be focused on underlying physiological and psychological issues causing infertility.

Endometrial tissue, as one of the key elements on ensuring successful embryo implantation and pregnancy, is a pertinent candidate for studies of mechanisms of infertility. It is bound to wide range of reproductive disorders causing infertility, such as endometriosis, a disease in which tissue that normally grows inside the uterus grows outside and causes chronic pelvic pain and infertility [17]; too thin < 7 mm or too thick > 12 mm endometrium in the luteal phase, lowering chances of pregnancy [18,19]; Asherman’s syndrome (AS) [20]; endometrial cancer [21]; and even polycystic ovarian syndrome (PCOS). PCOS, a frequent endocrine disorder, is defined by excess levels of circulating androgens and (or) hyperandrogenism followed by oligo/anovulation. Evidence shows that the metabolic and endocrinologic abnormalities observed in PCOS may affect the endometria and contribute to the infertility and endometrial disorders observed in females with this condition [22]; thus, they all have one thing in common: the exact mechanisms, or part of mechanisms of the pathogenesis of endometrium-associated diagnosis are unknown.

In the interest of determining the effects of reproductive disorders on genetic and epigenetic profile of endometrial tissue in this study, we analyzed the expression of decidualization related genes (*PRL*, *IGFBP1*, *WNT4*, *HOXA10*, *FOXO1*, *HAND2*, *ESR1*, *PGR*, *MUC1*, *MUC16*, *AXIN2*, *CSF2*, *TGFB1*, *TGFBR1*, *TP53*), angiogenesis related genes (*HIF1A*, *VEGFA*, *KDR*, *FLT4*, *PDGFA*, *PDGFRA*, *PDGFB*, *PDGFRB*), and immune response and inflammation-related genes (*NFKB1*, *NFKB2*, *REL*, *RELA*, *RELB*, *TNFA*, *TNFRSF1A*, *IL1B*, *IL2*, *IL18*, *INFG*, *CCL2*, *CCL5*, *CXCL1*, *CXCL10*). Furthermore, we investigated the expression of reproductive-system-related microRNAs: miR-34a, miR-125b and miR-223. Finally, we observed levels of decidualization-related proteins (Toll-like receptor 9, FoxO3a, NFKB, Akt, Phospho-Akt) and epigenetic-regulation-related proteins (H3K27me3, H3K4me3, H4K16Ac, HyperAcH4). Identified changes of molecular profile in endometrium of females with reproductive pathologies could enhance knowledge of molecular mechanisms substantial to endometrium-related disorders.

## 2. Materials and Methods

### 2.1. Patient Group

We recruited 25 females undergoing assisted reproductive technologies procedures–IVF/ICSI (in vitro fertilization/intracytoplasmic sperm injection) due to infertility at Vilnius University Hospital Santaros Klinikos Obstetrics and Gynecology Center Santaros Fertility Center from January to September of 2020. Inclusion criteria for control group endometrial tissue samples utilized in this study were females admitted for treatment due to male infertility. Inclusion criteria for pathology group were patients diagnosed with unexplained infertility, tubal factor infertility, and endometriosis. The collection and use of these samples were approved by the Ethics Committee of Biomedical Research of Vilnius District, No 158200-18/7-1049-550.

### 2.2. Endometrial Tissue Sample Preparation

Endometrial tissue samples were collected while performing endometrial scratching before IVF/ICSI approximately 7–9 days before the period was due. Endometrium biopsy was performed by specialists in reproductive medicine in accordance with local ethics policies and guidelines. To isolate RNA and proteins from endometrial tissue, the obtained specimen was transferred to a sterile Petri dish, washed 3 times with sterile PBS (Gibco, Thermo Fisher Scientific, Waltham, MA, USA), and cut with the sterile scalpel into 1–2 mm pieces. The cut specimen was transferred to thoroughly cleaned pestle, fast-frozen with liquid nitrogen, and ground to a consistency of a powder. Tissue powder was then mixed with 400 µL of DNA/RNA Lysis Buffer from Quick-DNA/RNA™ Miniprep Kit (Zymo Research, Irvine, CA, USA) followed by total RNA and protein purification according to manufacturer’s instructions.

### 2.3. Gene Expression Analysis by RT-qPCR

RT-qPCR samples were prepared using commercial kits, according to the manufacturer’s instructions. In brief: total RNA was purified using Quick-DNA/RNA™ Miniprep Kit (Zymo Research, Irvine, CA, USA), 250 ng of purified RNA was reverse transcribed to synthesize cDNA with oligo (dT) primers using FIREScript cDNA Synthesis Kit (Solis BioDyne, Tartu, Estonia), and qPCR was performed with gene specific primers using HOT FIREPol qPCR Mix (Solis BioDyne, Tartu, Estonia) with following conditions: activation 10 min 95 °C; 40 cycles of denaturation 10 s 95 °C; annealing 20 s with temperature optimal for each primer; extension 20 s 72 °C on the RotorGene 6000 system (Corbett Life Science, QIAGEN, Hilden, Germany). Primer sequences are presented in Table S1. mRNA levels were normalized to *GAPDH* expression. Relative gene expression was calculated using the ΔΔCt method.

### 2.4. MiRNA Analysis

RT-qPCR samples for miRNA analysis were prepared using commercial kits, according to the manufacturer’s instructions. Total RNA was purified using Quick-DNA/RNA™ Miniprep Kit (Zymo Research, Irvine, CA, USA), 5 ng of total RNA and 5X primers were used to synthesize individual cDNA for each miRNA. cDNA was synthesized using TaqMan™ MicroRNA Reverse Transcription Kit (Thermo Fisher Scientific, Waltham, MA, USA), and qPCR was performed with 20X primers using TaqMan™ Universal Master Mix II, no UNG Kit (Thermo Fisher Scientific, Waltham, MA, USA) with the following conditions: polymerase activation 10 min 95 °C; 40 cycles of denaturation 15 s 95 °C; annealing/extention 60 s 60 °C on the RotorGene 6000 system (Corbett Life Science, QIAGEN, Hilden, Germany). Primer sequences are presented in Table S2. MiRNA levels were normalized to RNU48 expression. Relative expression was calculated using the ΔΔCt method.

### 2.5. Protein Isolation and Immunoanalysis by Western Blot

Proteins from endometrial tissue samples were extracted parallel to RNA using Quick-DNA/RNA™ Miniprep Kit (Zymo Research, Irvine, CA, USA). Proteins were fractionated on a 7–15% polyacrylamide gradient SDS/PAGE gel using Tris-glycine buffer. The PVDF membrane (Immobilon P; Millipore, Billerica, MA, USA) were probed with the primary antibodies with dilutions: Beta-Actin, 1:500; Toll-like receptor 9, 1:1000; FoxO3a, 1:1000; NFκB, 1:200; Akt, 1:1000; Phospho-Akt, 1:1000; H3, 1:1000; H4, 1:1000; H3K27me3, 1:1000; H3K4me3, 1:2000; H4K16Ac, 1:2000; HypecAcH4, 1:1000; in accordance with the manufacturer’s instructions. The membrane was subsequently washed four times with PBS–Tween-20 and then incubated with horseradish-peroxidase (HRP)-linked secondary corresponding antibody for 1 h at room temperature. Βeta-Actin and histones H3 and H4 were used as loading controls. Chemiluminescent signal detection was carried out on ChemiDoc XRS+ System (BIORAD, Hercules, CA, USA). Quantitative evaluation was performed using ImageJ software.

### 2.6. Statistical Analysis

Statistical analysis was performed using GraphPad Prism version 8.0.1 for Windows, GraphPad Software, San Diego, California USA. Patient count: control group *n* = 11, pathology group *n* = 14. Data in graphs are represented as mean ± standard deviation (S.D.), and triangular data points indicate outliers determined by ROUT (Q = 5%). The statistical significance of difference of means of control and pathology groups was calculated using the Mann–Whitney U test; significance was set at *p* ≤ 0.05 (*), *p* ≤ 0.01 (**), and *p* ≤ 0.001 (***).

## 3. Results

### 3.1. Study Population and the Clinical Data

To analyze infertility-related molecular and epigenetic changes in endometrial tissue, 25 females undergoing IVF treatments were selected. Females with diagnosis of male infertility were considered control group (11 patients) and females with unexplained infertility, tubal factor infertility, or endometriosis were assigned to pathology group (14 patients). Clinical data of patients are represented in Table 1. Average maternal age and duration of infertility in the control group were 32.8 years old and 3.4 years, and in the pathology group, they were 33.4 years old and 4.4 years, respectively, and did not differ significantly (*p* = 0.7; *p* = 0.3). Although patient age and infertility duration in both groups were comparable, conception rate differed notably: in the control group, 90.9% of patients conceived, and in the pathology group, 64.3% patients conceived after IVF. Control patients were seen to have a successful pregnancy more often than patients with pathology (72.7% and 35.7%, respectively).

### 3.2. Changes of Decidualization, Angiogenesis, Immune Response, and Inflammation-Related Gene Expression in Endometrial Tissue

**Figure 1:**
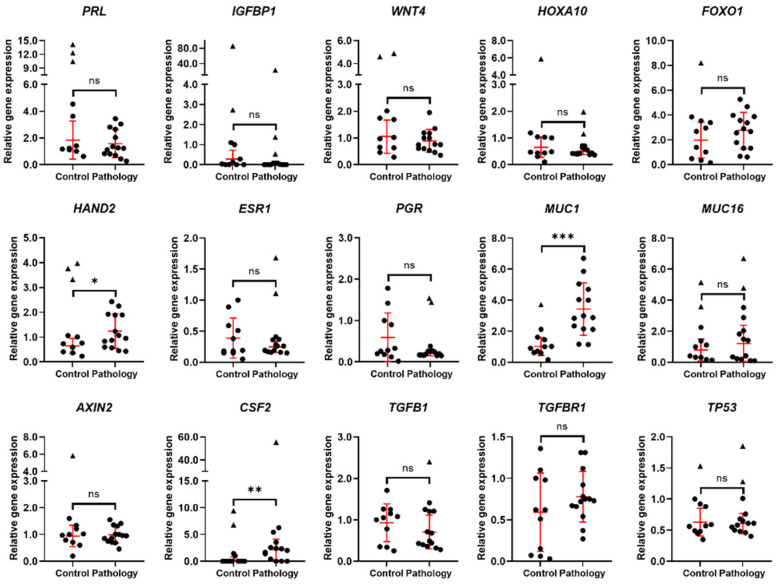
Expression of decidualization and implantation related genes. Results are presented as mean ± standard deviation (red lines), triangle data points represent outliers based on ROUT (Q = 5%). In the control group, *n* = 11; in the pathology group, *n* = 14. * *p* ≤ 0.05; ** *p* ≤ 0.01; *** *p* ≤ 0.001; ns—not statistically significant, based on the Mann–Whitney U test.

To target the effects of reproductive disorders on success of conception and pregnancy, the analysis of expression changes of 15 decidualization- and implantation-related genes was performed on endometrial tissue samples of control patients and patients with pathologies who successfully conceived or did not conceive (Figure 1). Expression of *PRL*, *IGFBP1*, *WNT4*, and *HOXA10* remained stable comparing control and pathology groups, pathology group possessed slightly higher expression of *FOXO1* and significantly higher expression of *HAND2*; however, expression of *ESR1* and *PGR* was slightly decreased compared to the control group. *MUC1* and *CSF2* was significantly upregulated in endometrial tissue of females with pathology; on the other hand, reproductive pathologies had no compelling effect on the expression of *MUC16*, *AXIN2*, *TGFB1*, *TGFBR1*, and *TP53.* The obtained results suggest that reproductive pathologies may affect endometrial tissue capability of decidualization, possibly having impact on chances of conception and maintenance of pregnancy.

**Figure 2:**
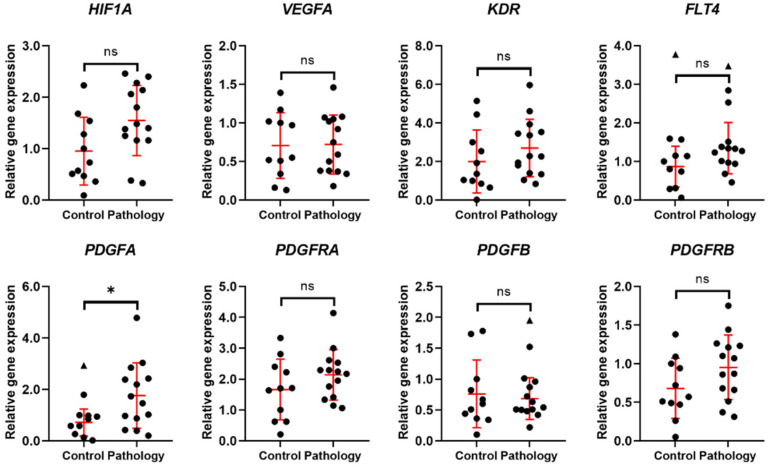
Expression of angiogenesis-related genes. Results are presented as mean ± standard deviation (red lines); triangle data points represent outliers based on ROUT (Q = 5%). In the control group, *n* = 11; in the pathology group, *n* = 14. * *p* ≤ 0.05; ns—not statistically significant, based on the Mann–Whitney U test.

In comparison to the control group, endometrium of patients with reproductive pathologies retains slightly higher expression of *HIF1A*, *KDR*, and *FLT4* and unchanged expression of *VEGFA* (Figure 2). Moreover, in the pathology group, *PDGFA* is significantly upregulated and its receptor PDGFRA tends to be upregulated as well. Expression of *PDGFB* remains stable in both study groups, while expression of *PDGFRB* tends to be higher in the pathology group. Studies of the expression of 8 genes related to angiogenesis have shown that reproductive pathologies may have an impact on molecules of the angiogenesis pathway.

**Figure 3:**
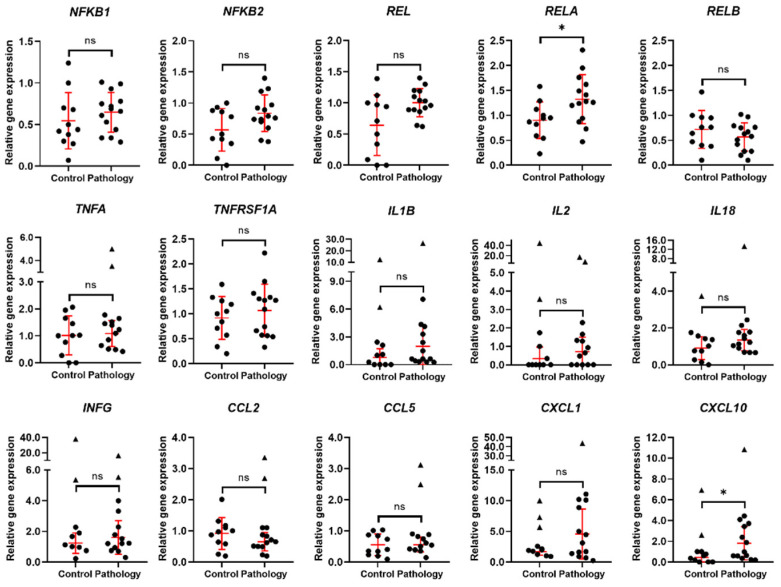
Expression of immune response and inflammation-related genes. Results are presented as mean ± standard deviation (red lines); triangle data points represent outliers based on ROUT (Q = 5%). In the control group, *n* = 11; in the pathology group, *n* = 14. * *p* ≤ 0.05; ns—not statistically significant, based on the Mann–Whitney U test.

During the analysis of the expression of the 15 major genetic markers of immune response and inflammation in endometrial tissue, it was shown that the expression of genes, coding monomers of nuclear factor kappa B (NFκB), *NFKB1*, *NFKB2*, *REL*, and *RELA* was increased; however, the expression of *RELB* was slightly decreased in the endometrium of patients of the pathology group (Figure 3). Although the expression of *TNFA1* and its receptor gene *TNFRSF1A* was stable in both groups, genes coding interleukins (*IL1B*, *IL2*, *IL18*) and *INFG* tend to be increased in the endometrium of females with pathologies. The expression of *CCL2* was slightly higher in the endometrium of the control group, and expression of *CCL5* was stable in both female groups. Genes *CXCL1* and *CXCL10* were upregulated in the endometria of the pathology group compared to the control group. Considering that most of the immune response and inflammation-related genes imply increase of expression in endometrium of pathology group, it draws to conclusion that endometrial tissue of females with reproductive pathologies might be in a state of enlarged inflammation, affecting its reproductive function.

### 3.3. MiRNAs in Endometrial Tissue

**Figure 4:**
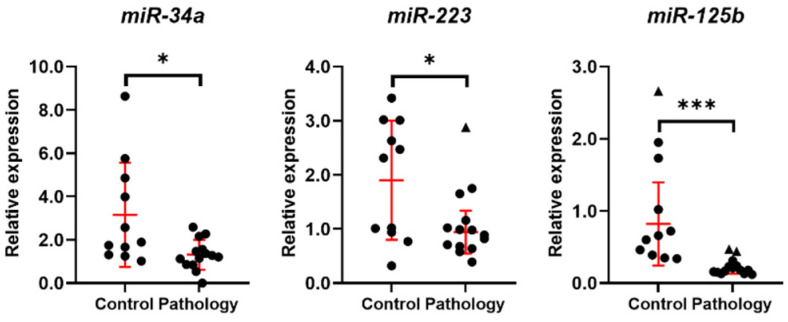
Expression of miRNA related to regulation of genes of reproduction system cells. Results are presented as mean ± standard deviation (red lines); triangle data points represent outliers based on ROUT (Q = 5%). In the control group, *n* = 11; in the pathology group, *n* = 14. * *p* ≤ 0.05; *** *p* ≤ 0.001, based on the Mann–Whitney U test.

Since miRNAs play an important role in posttranscriptional gene regulation, in this study, the expression of three reproduction-system-related miRNAs was investigated (Figure 4). It was shown that expression of miR-34a-3p, miRNA-223-5p, and miRNA-125b-5p is significantly lower in endometrial tissue of patients with reproductive pathologies compared to endometrial tissue from females of the control group.

### 3.4. Protein Profile Changes in Endometrial Tissue

**Figure 5:**
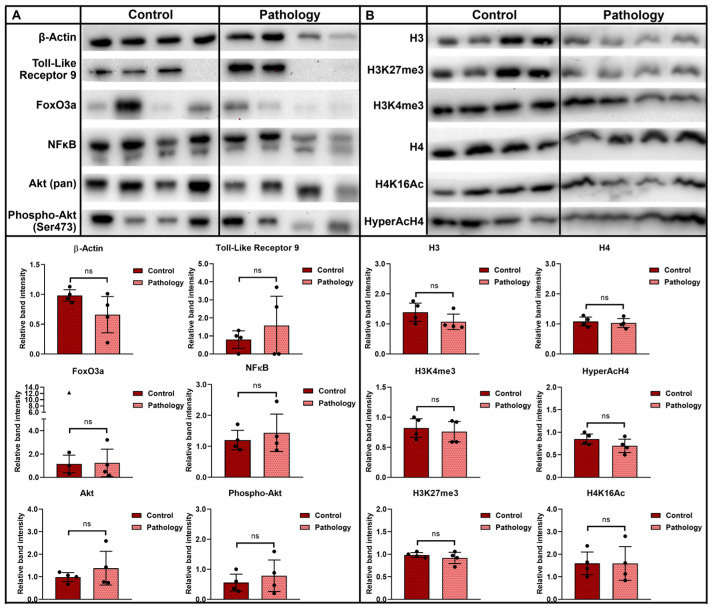
Evaluation of changes in protein levels in the control group and in the pathology group *n* = 4. (**A**) Proteins of decidualization pathways. (**B**) Histone modifications. Beta-Actin and histones H3 and H4 are control proteins. The intensity of the protein bands was estimated by the ImageJ program, and the levels of each protein were calculated according to the Beta-Actin, Akt, H3, and H4. Densitometric analysis graphs of relative band intensity of detected protein levels was measured using ImageJ software and normalized to the loading control. Results are presented as mean ± standard deviation; triangle data points represent outliers based on ROUT (Q = 5%); ns—not statistically significant, based on the Mann–Whitney U test.

In this study, proteins involved in decidualization Toll-like receptor 9, FoxOa3, NFκB, Akt, Phospho-Akt (Figure 5A), and epigenetic regulation H3K27me3, H3K4me3, H4K16Ac, HyperAcH4 (Figure 5B) were analyzed using Western blot, and densitometric analysis graphs of relative band intensity of detected protein levels were created from measurements and presented. The levels of Toll-like receptor 9 and FoxO3a proteins in the endometrial tissue of control group females and pathology group were found to remain stable, whereas levels of NFκB, Akt, and Phospho-Akt tend to show slight increases in the endometria of patients with reproductive pathologies. Levels of epigenetic regulation proteins H3K27me3, H3K4me3, and H4K16Ac remained unchanged comparing tissues of control patients and patients with pathologies, while HyperAcH4 appears to be downregulated in the pathology group. Analysis of protein levels suggests that increases of NFκB levels in endometrium of pathology group females, though not significant, correlate with gene expression analysis and support the hypothesis of inflammation of endometrium possibly being relevant to reproduction outcome.

## 4. Discussion

The endometrium has a significant role in reproduction since the physiological functions of the endometrium are preparation for implantation, maintenance of pregnancy before the formation of placenta, and in the lack of pregnancy-menstruation [23]. Failure of the endometrium biochemical, hormonal, and immunological decidualization in preparation for the advancing of blastocyst leads to abnormal implantation/placentation and finally to adverse pregnancy outcome. This process includes the downregulation of the expression of genes of the proinflammatory response, alongside the upregulation of genes that promote immune tolerance, stimulates angiogenesis, and enables tissue invasion [24]. In this research, we investigated endometrial tissue in the luteal phase when endometrium should be the most receptive for embryo implantation. In the first part of the investigation, we compared the expression profile of decidualization-related genes in endometrium of control females and females with reproductive pathology. To begin with, we showed that endometria from patients with reproductive pathology possess upregulated forkhead box O1A (*FOXO1)* and significantly upregulated heart and neural crest derivatives–expressed transcript 2 (*HAND2).* Su et al. (2014) showed that Notch1 modulates multiple signaling mechanisms critical for decidualization and *FOXO1* acts as a downstream target of Notch signaling. In some disorders, for example, endometriosis is associated with decreased expression of *NOTCH1*-regulated, *FOXO1*-responsive genes during decidualization [25]. HAND2 is known to be a major transcription factor in the progestin-induced decidualization process. HAND2 was suggested to be able to regulate interleukin 15 (IL15), a major immune factor involved in the activation and survival of uterine natural killer (uNK) cells. Activated uNK cells can direct the migration and invasion of the trophoblasts by produce angiogenic factors and secrete cytokines [26]. It was shown that E2 + MPA increases *HAND2* mRNA levels in endometrium stem cells in a time- and dose-dependent manner [27]. Increased expression of *HAND2* could suggest more prominent inflammation and immune response in endometrium of pathology group females. Furthermore, we showed that expression of *ESR1* and *PGR* was slightly decreased in endometrium of pathology group compared to control group. Decidualization is a steroid-hormone-regulated process. The progesterone receptor (PGR) and estrogen receptors (ESR1 and ESR2) induce P4 and E2-responsive signaling pathways, regulated in an epithelial and stromal compartment-specific manner in the endometrium. In case of a loss of epithelial-stromal P4 and E2 signaling, P4 resistance and E2 dominance are likely to develop, potentially leading to uterine disorders such as endometriosis [28]. In our research, *MUC1* and *CSF2* was significantly upregulated in endometrial tissue of females with reproductive pathology. Loss of *MUC1* at implantation sites is shown to be necessary for embryo attachment and implantation. The expression of *MUC1* is regulated by progesterone and proinflammatory cytokines, such as *TNFα* and *IFNγ*. Then, MUC1 expression is high, and proinflammatory cytokines are highly expressed in uterine tissues, thus activating *MUC1* expression through their downstream transcription factors [29]. In the decidua, neutrophils and macrophages, essential for basal state of inflammation in pregnancy, are activated by Colony Stimulating Factor 2 (CSF2). Arcuri et al. (2009) showed that CSF2 is highly expressed in decidua from patients with chorioamnionitis and indicated *TNF* or *IL1B* as important regulators of decidual leukocyte infiltration and activation [30]. Data suggest that increased expression of *MUC1* could have role in decrease of implantation potential in endometrium of patients with reproductive pathologies.

Studying angiogenesis-related gene expression, we discovered that in comparison to the control group, the endometria of pathology group females retain mostly higher expression of genes of angiogenesis. In patients with pathology, *PDGFA* is significantly upregulated and its receptor *PDGFRA* tends to be upregulated as well. It is known that vascular development occurs during embryo implantation and early placentation of normal pregnancies [31]. In 2012, evidence that *MUC1* regulates the expression and secretion of platelet-derived growth factor-A (PDGFA), proangiogenic cytokine, was shown: Muc1 (Mucin 1) associates with Hif1-α (Hypoxia-inducible factor), a transcription factor that is involved in *PDGFA* expression regulation [32]. Since our data showed increases in both expression of *MUC1* and *PDGFA* in pathology group, it could support the hypothesis of expression control. It is shown that remodeling of the vascular structure of maternal endometrium is required for the accommodation of the rapidly growing embryo. Importance of angiogenesis was proved by showing that inhibition of angiogenesis with an antiangiogenic compound interrupts placentation in mice and results in resorption of all embryos [31].

Implantation has been characterized as an inflammatory-type response. Multiple types of immune cells play a pivotal role in the early and late steps of embryo implantation as well as in placentation including neutrophils, monocytes, and macrophages, mast cells, uterine natural killer cells (uNK), dendritic cells, B cells, and T cells. A number of cytokines have been identified at the implantation site [33,34]. In the midsecretory phase and early pregnancy, the number of decidual natural killer (dNK) cells increases. During pregnancy, they secrete cytokines and growth factors (IL-10, TNF-γ, PlGF, IL-1, GM-CSF, TGF-1, CSF-1, LIF, and IFN-γ) and angiogenic factors, such as angiopoietin-2 and VEGF, thus helping the trophoblast migration [35]. While analyzing the expression of the 15 major genetic markers of immune response and inflammation in endometrial tissue, we showed that the expression of genes coding inflammation-related molecules is generally increased in endometrium of females with reproductive pathology. Expression of *NFKB1*, *NFKB2*, *REL*, *RELA*, interleukins (*IL1B*, *IL2*, *IL18*), *INFG*, *CCL2*, *CXCL1*, and *CXCL10* is increased in the endometria of the pathology group. NFκB is involved in signaling by modulating DNA transcription, thereby regulating essential immune responses [36]. Studies show that during implantation, the expression and secretion of inflammatory mediators, such as IFNG and IFND, PGF2α and PGE2, TNFA, IL1B, IL6, IL11, and LIF are increased by the endometrium [37]. Success of implantation after in vitro fertilization has been linked to high concentrations of IL1A and IL1B in the culture medium of human embryos. IL1B is shown to participate in implantation by indirectly increasing concentration of intracellular cAMP by upregulating PTGS2 and PGE2, thus reorganizing the actin cytoskeleton [33]. It is demonstrated that in the mid-to-late-secretory phase endometrium, where implantation occurs, and the concentrations of ovarian steroid hormones are highest, high expression levels of cytokines such as LIF and IL6 are detected [38]. Immune cells, such as dNK, play a role in pathological pregnancy and are related to preeclampsia, recurrent pregnancy loss, endometriosis, and recurrent implantation failure (RIF). It was shown that the number of cytotoxic uNK cell-surface receptors on uNK cells and increased number of immature uNK cells was upregulated in the endometrium of infertile females with endometriosis, compared to fertile females. Moreover, NKp44 expression on uNK cells was significantly increased in RIF patients and indicated that NK cells’ cytotoxicity may cause the recurrent implantation failure [39,40,41].

In previous studies of various scientific groups, it has been determined that thousands of protein coding genes change their expression levels in the endometrium throughout the menstrual cycle. Mechanisms of epigenetic regulation, such as DNA methylation, noncoding RNAs and histone post-translational modifications induce the expression of genes associated with stromal cell proliferation, endometrial epithelial growth, transcriptional regulation, and angiogenesis. During decidualization and implantation, epigenetic remodeling occurs in epithelial and stromal cells, leading to rearrangement of gene expression and cell function [42]. In this study, we showed that expression of miR-34a, miRNA-223, and miRNA-125b is significantly lower in endometrial tissue of patients with reproductive pathologies compared to endometrial tissue from control females. Studies show that miRNA have significant role in formation of many reproduction-related disorders. MiRNA-34a is known to be involved in the pathophysiology of preeclampsia [43]. Moreover, miR-34a-5p can temporarily inhibit VEGF during ovulation by regulation TGFB1 [44]. Quin et al. in 2019 showed that miR-223 that is related to immune tolerance and inflammatory response is a main factor in Dicer-regulated adipose differentiation, and the dysfunction of Dicer could be important in obesity of PCOS patients [45]. miRNA-233 is generally found to be downregulated in preeclampsia, both in placental and circulatory samples [46]. Expression of miRNA-125b is shown to be increased in patients with endometriosis [47]. Nevertheless, in patients with epithelial ovarian cancer (EOC) serum levels of miR-125b were significantly lower than that of other controls and was associated with tumor development, progression, metastasis, and poor prognosis [48].

Histone modifications play an important role in the formation of DNA structure, interaction of transcription factors, and the regulation of gene expression [49]. In this study, we demonstrated that levels of epigenetic regulation proteins H3K27me3, H3K4me3, and H4K16Ac remained stable comparing tissues of the control group and the pathology group, while HyperAcH4 appears to be downregulated in patients with reproductive pathology. It is known that bivalent histone modifications—H3 lysine 27 trimethylation (H3K27me3) and H3 lysine 4 trimethylation (H3K4me3)—are important for the regulation of lineage control genes’ expression. H3K27me3 mark is detected when lineage-regulatory genes are repressed during stem cell pluripotency and the H3K4me3 mark is detected in the active chromatin regions during cell differentiation [49]. A 2018 study by Monteiro et al. showed that the acetylation level of histone H4 on lysine 16 (H4K16ac) was decreased in endometriosis samples, the methylation levels of histone H3 on lysine 9 (H3K9me) was lower in control endometrium in comparison to endometriosis, and the average of total methylation level of histone H3 on lysine 27 (H3K27me) was significantly increased in patients with endometriosis [50].

We believe that this study could be beneficial for investigation of universal markers for infertility of unknown origin in females, although to apply this information to clinical studies, sample size should be vastly enlarged. Following studies could be focused on investigation of endometrium tissue to look for molecular markers of infertility of unknown origin and, moreover, search for molecular markers in endometrium of females with specific diagnosis—for example, endometriosis or tubal factor infertility. Target molecules for such research could be related to process of inflammation and regulation of cellular metabolism.

## 5. Conclusions

Molecular causes of infertility, though widely studied in recent decades, still remain unsolved. In this research, we investigated the genetic profile of endometrial tissue from females admitted for treatment due to male infertility and females with reproductive pathologies who are undergoing assisted reproductive technologies procedures including unexplained infertility, infertility due to endometriosis, and tubal factor infertility. It was demonstrated that expression of studied immune response related genes is higher, and levels of miR-34a-3p, miRNA-223-5p, and miRNA-125b-5p is lower in the endometria of females with reproductive pathologies. According to the results, we suggest that the studied reproductive pathologies could be associated to the inflammatory processes and be accompanied by epigenetic changes. These results could be the background for further studies in searching effective diagnosing and treatment strategies for infertile couples.

## Figures and Tables

**Table 1:** Clinical characteristics of the study population.

Characteristics	Control	Pathology
Patient count (*n*)	11	14
Maternal age (years)	32.8 ± 4.0	33.4 ± 4.0
Duration of infertility (years)	3.4 ± 2.4	4.4 ± 2.4
Conceived (*n*)	10	9
Successful pregnancy (*n*)	8	5
BMI > 25 (*n*)	3	0
Hormone level abnormalities (*n*)	1	3
Diagnosis (*n*)	Male infertility (11)	Tubal factor infertility (4) Unexplained infertility (10)
Endometriosis (*n*)	0	3

*n*, number of females possessing characteristic; BMI, body mass index; hormone level abnormalities, prolactinemia, hipotyrosis.

## Data Availability

The data presented in this study are available on request from the corresponding author.

## Supplementary Materials

The following supporting information can be downloaded at: https://www.mdpi.com/article/10.3390/biomedicines10061324/s1, Table S1: Primers used for RT-qPCR gene expression analysis; Table S2: Primers used for RT-qPCR miRNA expression analysis.

## References

[1] Zhang S., Lin H., Kong S., Wang S., Wang H., Wang H., Armant D.R. (2013). Physiological and molecular determinants of embryo implantation. Mol. Aspects Med..

[2] Liang J., Wang S., Wang Z. (2017). Role of MicroRNAs in embryo implantation. Reprod. Biol. Endocrinol..

[3] Teh W.-T., McBain J., Rogers P. (2016). What Is the contribution of embryo-endometrial asynchrony to implantation failure?. J. Assist. Reprod. Genet..

[4] Lessey B.A., Young S.L. (2019). What exactly is endometrial receptivity?. Fertil. Steril..

[5] Harper M.J. (1992). The implantation window. Baillieres Clin. Obstet. Gynaecol..

[6] Navot D., Scott R.T., Droesch K., Veeck L.L., Liu H.C., Rosenwaks Z. (1991). The window of embryo transfer and the efficiency of human conception in vitro. Fertil. Steril..

[7] Ashary N., Tiwari A., Modi D. (2018). Embryo implantation: War in times of love. Endocrinology.

[8] Gellersen B., Brosens J.J. (2014). Cyclic decidualization of the human endometrium in reproductive health and failure. Endocr. Rev..

[9] Kim J.J. (2021). Preparing for implantation. eLife.

[10] Okada H., Tsuzuki T., Murata H. (2018). Decidualization of the human endometrium. Reprod. Med. Biol..

[11] Mrozikiewicz A.E., Ożarowski M., Jędrzejczak P. (2021). Biomolecular markers of recurrent implantation failure—A review. Int. J. Mol. Sci..

[12] Kamphuis E.I., Bhattacharya S., van der Veen F., Mol B.W.J., Templeton A. (2014). Are we overusing IVF?. BMJ.

[13] Duffy J.M.N., Adamson G.D., Benson E., Bhattacharya S., Bhattacharya S., Bofill M., Brian K., Collura B., Curtis C., Evers J.L.H. (2021). Top 10 priorities for future infertility research: An international consensus development study. Fertil. Steril..

[14] Sullivan-Pyke C.S., Senapati S., Mainigi M.A., Barnhart K.T. (2017). In vitro fertilization and adverse obstetric and perinatal outcomes. Semin. Perinatol..

[15] Namdar A., Naghizadeh M.M., Zamani M., Yaghmaei F., Sameni M.H. (2017). Quality of life and general health of infertile women. Health Qual. Life Outcomes.

[16] Vaughan D.A., Shah J.S., Penzias A.S., Domar A.D., Toth T.L. (2020). Infertility remains a top stressor despite the COVID-19 pandemic. Reprod. Biomed. Online.

[17] Kim T.H., Yoo J.-Y., Choi K.-C., Shin J.-H., Leach R.E., Fazleabas A.T., Young S.L., Lessey B.A., Yoon H.-G., Jeong J.-W. (2019). Loss of HDAC3 results in nonreceptive endometrium and female infertility. Sci. Transl. Med..

[18] Zhang J., Sun Y.-F., Xu Y.-M., Shi B., Han Y., Luo Z.-Y., Zhao Z.-M., Hao G.-M., Gao B.-L. (2021). Effect of endometrium thickness on clinical outcomes in luteal phase short-acting GnRH-a long protocol and GnRH-ant protocol. Front. Endocrinol..

[19] Liu K.E., Hartman M., Hartman A. (2019). Management of thin endometrium in assisted reproduction: A clinical practice guideline from the Canadian fertility and andrology society. Reprod. BioMed. Online.

[20] Dreisler E., Kjer J.J. (2019). Asherman’s syndrome: Current perspectives on diagnosis and management. Int. J. Womens Health.

[21] Urick M.E., Bell D.W. (2019). Clinical actionability of molecular targets in endometrial cancer. Nat. Rev. Cancer.

[22] Giudice L.C. (2006). Endometrium in PCOS: Implantation and predisposition to endocrine CA. Best Pract. Res. Clin. Endocrinol. Metab..

[23] Critchley H.O.D., Maybin J.A., Armstrong G.M., Williams A.R.W. (2020). Physiology of the endometrium and regulation of menstruation. Physiol. Rev..

[24] Ng S.-W., Norwitz G.A., Pavlicev M., Tilburgs T., Simón C., Norwitz E.R. (2020). Endometrial decidualization: The primary driver of pregnancy health. Int. J. Mol. Sci..

[25] Su R.-W., Strug M.R., Joshi N.R., Jeong J.-W., Miele L., Lessey B.A., Young S.L., Fazleabas A.T. (2015). Decreased notch pathway signaling in the endometrium of women with endometriosis impairs decidualization. J. Clin. Endocrinol. Metab..

[26] Murata H., Tanaka S., Tsuzuki-Nakao T., Kido T., Kakita-Kobayashi M., Kida N., Hisamatsu Y., Tsubokura H., Hashimoto Y., Kitada M. (2020). The transcription factor HAND2 Up-regulates transcription of the IL15 gene in human endometrial stromal cells. J. Biol. Chem..

[27] Cho H., Okada H., Tsuzuki T., Nishigaki A., Yasuda K., Kanzaki H. (2013). Progestin-induced heart and neural crest derivatives expressed transcript 2 is associated with Fibulin-1 expression in human endometrial stromal cells. Fertil. Steril..

[28] Marquardt R.M., Kim T.H., Shin J.-H., Jeong J.-W. (2019). Progesterone and estrogen signaling in the endometrium: What goes wrong in endometriosis?. Int. J. Mol. Sci..

[29] Dharmaraj N., Wang P., Carson D.D. (2010). Cytokine and progesterone receptor interplay in the regulation of MUC1 gene expression. Mol. Endocrinol..

[30] Arcuri F., Toti P., Buchwalder L., Casciaro A., Cintorino M., Schatz F., Rybalov B., Lockwood C.J. (2009). Mechanisms of leukocyte accumulation and activation in chorioamnionitis: Interleukin 1β and tumor necrosis factor α enhance colony stimulating factor 2 expression in term decidua. Reprod. Sci..

[31] Torry D.S., Leavenworth J., Chang M., Maheshwari V., Groesch K., Ball E.R., Torry R.J. (2007). Angiogenesis in implantation. J. Assist. Reprod. Genet..

[32] Sahraei M., Roy L.D., Curry J.M., Teresa T.L., Nath S., Besmer D., Kidiyoor A., Dalia R., Gendler S.J., Mukherjee P. (2012). MUC1 regulates PDGFA expression during pancreatic cancer progression. Oncogene.

[33] Geisert R., Fazleabas A., Lucy M., Mathew D. (2012). Interaction of the conceptus and endometrium to establish pregnancy in mammals: Role of interleukin 1β. Cell Tissue Res..

[34] Schumacher A., Zenclussen A.C. (2019). Human chorionic gonadotropin-mediated immune responses that facilitate embryo implantation and placentation. Front. Immunol..

[35] Mahajan D., Sharma N.R., Kancharla S., Kolli P., Tripathy A., Sharma A.K., Singh S., Kumar S., Mohanty A.K., Jena M.K. (2022). Role of natural killer cells during pregnancy and related complications. Biomolecules.

[36] Sfakianoudis K., Rapani A., Grigoriadis S., Pantou A., Maziotis E., Kokkini G., Tsirligkani C., Bolaris S., Nikolettos K., Chronopoulou M. (2021). The role of uterine natural killer cells on recurrent miscarriage and recurrent implantation failure: From pathophysiology to treatment. Biomedicines.

[37] Goryszewska-Szczurek E., Baryla M., Kaczynski P., Waclawik A. (2021). Prokineticin 1–prokineticin receptor 1 signaling in trophoblast promotes embryo implantation and placenta development. Sci. Rep..

[38] Park H., Cho M., Do Y., Park J.-K., Bae S.-J., Joo J., Ha K.-T. (2021). Autophagy as a therapeutic target of natural products enhancing embryo implantation. Pharmaceuticals.

[39] Zhang X., Wei H. (2021). Role of decidual natural killer cells in human pregnancy and related pregnancy complications. Front. Immunol..

[40] Giuliani E., Parkin K.L., Lessey B.A., Young S.L., Fazleabas A.T. (2014). Characterization of uterine NK cells in women with infertility or recurrent pregnancy loss and associated endometriosis. Am. J. Reprod. Immunol..

[41] Thiruchelvam U., Wingfield M., O’Farrelly C. (2016). Increased UNK progenitor cells in women with endometriosis and infertility are associated with low levels of endometrial stem cell factor. Am. J. Reprod. Immunol..

[42] Retis-Resendiz A.M., González-García I.N., León-Juárez M., Camacho-Arroyo I., Cerbón M., Vázquez-Martínez E.R. (2021). The role of epigenetic mechanisms in the regulation of gene expression in the cyclical endometrium. Clin. Epigenetics.

[43] Doridot L., Houry D., Gaillard H., Chelbi S.T., Barbaux S., Vaiman D. (2014). MiR-34a expression, epigenetic regulation, and function in human placental diseases. Epigenetics.

[44] Yerushalmi G.M., Salmon-Divon M., Ophir L., Yung Y., Baum M., Coticchio G., Fadini R., Mignini-Renzini M., Dal Canto M., Machtinger R. (2018). Characterization of the MiRNA regulators of the human ovulatory cascade. Sci. Rep..

[45] Qin L., Chen J., Tang L., Zuo T., Chen H., Gao R., Xu W. (2019). Significant role of dicer and MiR-223 in adipose tissue of polycystic ovary syndrome patients. Biomed. Res. Int..

[46] Sheikh A.M., Small H.Y., Currie G., Delles C. (2016). Systematic review of micro-RNA expression in Pre-eclampsia identifies a number of common pathways associated with the disease. PLoS ONE.

[47] Moustafa S., Burn M., Mamillapalli R., Nematian S., Flores V., Taylor H.S. (2020). Accurate diagnosis of endometriosis using serum MicroRNAs. Am. J. Obstet. Gynecol..

[48] Chen Z., Guo X., Sun S., Lu C., Wang L. (2020). Serum MiR-125b levels associated with epithelial ovarian cancer (EOC) development and treatment responses. Bioengineered.

[49] Vastenhouw N.L., Schier A.F. (2012). Bivalent histone modifications in early embryogenesis. Curr. Opin. Cell Biol..

[50] Monteiro J.B., Colón-Díaz M., García M., Gutierrez S., Colón M., Seto E., Laboy J., Flores I. (2014). Endometriosis is characterized by a distinct pattern of histone 3 and histone 4 lysine modifications. Reprod. Sci..

