# Supplementary material for: Characterization of Epigenetic and Molecular Factors in Endometrium of Females with Infertility
Source: Biomedicines. 2022 Jun 4;10(6):1324. doi: 10.3390/biomedicines10061324 (PMC9219839; doi:10.3390/biomedicines10061324)
Supplement: Supplementary file 1 [file biomedicines-10-01324-s001.zip › biomedicines-1738071-supplementary.pdf]

Table S1. Primers used for RT-qPCR gene expression analysis

Gene name	Sequence of forward and reverse primers (5' –> 3')	Product length (bp)
AXIN2	F: GACGGACAGCAGTGTAGATG<br>R: GGGTTCTCGGGAAATGA	125
CCL2	F: GCTCAGCCAGATGCAATCA<br>R: TTTGCTTGTCCAGGTGGTC	198
CCL5	F: TCATTGCTACTGCCCTCTGCG<br>R: CTCATCTCCAAAGAGTTGATG	214
CSF2	F: CACTGCTGCTGAGATGAATGAAA<br>R: GTCTGTAGGCAGGTCGGCTC	78
CXCL1	F: GGAACAGAAGAGGAAAGAGAGAC<br>R: TAGGACAGTGTGCAGGTAGA	109
CXCL10	F: GTGGCATTCAAGGAGTACCTC<br>R: TGATGGCCTTCGATTCTGGATT	223
ESR1	F: GGAATGATGAAAGGTGGGATACGA<br>R: GGCAGCTCTCATGTCTCCAG	123
FLT4	F: TGCACGAGGTACATGCCAAC<br>R: GCTGCTCAAAGTCTCTCACGAA	120
FOXO1	F: GGATGTGCATTCTATGGTGTACC<br>R: TTTCGGGATTGCTTATCTCAGAC	86
GAPDH	F: AGTCCCTGCCACACTCAG<br>R: TACTTTATTGATGGTACATGACAAGG	123
HAND2	F: ATGAGTCTGGTAGGTGGTTTTCC<br>R: CATACTCGGGGCTGTAGGACA	205
HIF1A	F: CCAACAGTAACCAACCTCAG<br>R: TCCTGTGGTGACTTGTCCTT	302
HOXA10	F: CAACTGGCTCACGGCAAAGA<br>R: TTCAGTTTCATCCTGCGGTTC	192
IGFBP1	F: TTGGGACGCCATCAGTACCTA<br>R: TTGGCTAAACTCTCTACGACTCT	114
IL18	F: CAACAAACTATTTGTCGCAGGA<br>R: TGCCACAAAGTTGATGCAAT	64
IL1B	F: AGATGATAAGCCCACTCTACAG<br>R: ACATTCAGCACAGGACTCTC	276
IL2	F: ATGAGACAGCAACCATTGTAGAATTT<br>R: CACTTAATTATCAAGTCAGTGTTGAGATGA	87
INFG	F: AGGGAAGCGAAAAAGGAGTCA<br>R: GGACAACCATTACTGGGATGCT	244
KDR	F: ACTGTCATCCTTACCAATCC<br>R: CCTCCAACTGCCAATACC	202
LDHA	F: ATGGCAACTCTAAAGGATCAGC<br>R: CCAACCCCAACAACTGTAATCT	86
MEF2C	F: GCCCTGAGTCTGAGGACAAG<br>R: AGTGAGCTGACAGGGTTGCT	163
MUC1	F: AGACGTCAGCGTGAGTGATG<br>R: CAGCTGCCCGTAGTTCTTTC	173
MUC16	F: AGCATCCTGGACGTAACCAC<br>R: CAGGTGGAAGGGTGTTCTGT	238
NFKB1	F: GAAGCACGAATGACAGAGGC<br>R: GCTTGGCGGATTAGCTCTTTT	137
NFKB2	F: ATGGAGAGTTGCTACAACCCA<br>R: CTGTTCCACGATCACCAGGTA	135

PDGFA	F: CACACCTCCTCGCTGTAGTATTTA<br>R: GTTATCGGTGTAAATGTCATCCAA	220
PDGFB	F: CTCGATCCGCTCCTTTGATGA<br>R: CGTTGGTGCGGTCTATGAG	239
PDGFRA	F: TTGAAGGCAGGCACATTTACA<br>R: GCGACAAGGTATAATGGCAGAAT	119
PDGFRB	F: AGACACGGGAGAATACTTTTGC<br>R: AGTTCCTCGGCATCATTAGGG	126
PGR	F: ATGGAAGGGCAGCACAACTAC<br>R: TTCTAAGGCGACATGCTGGG	94
PRL	F: GGAGCAAGCCCAACAGATGAA<br>R: GGCTCATTCCAGGATCGCAAT	75
REL	F: AAAGACTGCAGAGACGGCTA<br>R: CTCACCACATTGAGGTCACA	203
RELA	F: ATGTGGAGATCATTGAGCAGC<br>R: CCTGGTCCTGTGTAGCCATT	151
RELB	F: CCATTGAGCGGAAGATTCAACT<br>R: CTGCTGGTCCCGATATGAGG	122
TGFB1	F: GGGCTACCATGCCAACTTCT<br>R: GACACAGAGATCCGCAGTCC	384
TGFBR1	F: CACAGAGTGGGAACAAAAAGGT<br>R: CCAATGGAACATCGTCGAGCA	143
TNFA	F: GCTGCACTTTGGAGTGATCG<br>R: TCACTCGGGGTTCGAGAAGA	108
TNFRSFA1	F: TCACCGCTTCAGAAAACCACC<br>R: GGTCCACTGTGCAAGAAGAGA	96
TP53	F: TAACAGTTCCTGCATGGGCGGC<br>R: AGGACAGGCACAAACACGCACC	121
VEGFA	F: AGGGCAGAATCATCACGAAGT<br>R: AGGGTCTCGATTGGATGGCA	75
WNT4	F: CTCCACACTCGACTCCTTGC<br>R: CCGAAGAGATGGCGTACACG	86

Table S2. Primers used for RT-qPCR miRNA expression analysis

TaqMan™ MicroRNA Assay

Name	Accession Number	Sequence	Manufacturer
RNU48	NR_002745	GATGACCCCAGGTAACTCTGAGT GTGTCGCTGATGCCATCACCGCA GCGCTCTGACC	Thermo Fisher Scientific, Waltham, MA, USA
has-miR-34a-3p	MI0000268	CAAUCAGCAAGUAUACUGCCCU	Thermo Fisher Scientific, Waltham, MA, USA
hsa-miR-125b-5p	MI0000446	UCCCUGAGACCCUAACUUGUGA	Thermo Fisher Scientific, Waltham, MA, USA
hsa-miR-223-5p	MI0000300	UGUCAGUUUGUCAAAUACCCCA	Thermo Fisher Scientific, Waltham, MA, USA
